# Concordance between two monoclonal antibody-based antigen detection enzyme-linked immunosorbent assays for measuring cysticercal antigen levels in sera from pigs experimentally infected with *Taenia solium* and *Taenia hydatigena*

**DOI:** 10.1186/s13071-024-06197-6

**Published:** 2024-04-02

**Authors:** Gianfranco Arroyo, Luz Toribio, Sara Garrido, Nancy Chile, Teresa Lopez-Urbina, Luis A. Gomez-Puerta, Miguel Muro, Robert H. Gilman, Yesenia Castillo, Pierre Dorny, Seth E. O’Neal, Armando E. Gonzalez, Hector H. Garcia, for The Cysticercosis Working Group in Peru (CWGP)

**Affiliations:** 1 Center for Global Health, Universidad Peruana Cayetano Heredia, Lima, Peru; 2 Direccion General de Investigacion, Desarrollo e Innovacion, Universidad Cientifica del Sur, Lima, Peru; 3 School of Veterinary Medicine, Universidad Nacional Mayor de San Marcos, Lima, Peru; 4 Laboratory of Infectious Diseases, Faculty of Sciences and Philosophy, Universidad Peruana Cayetano Heredia, Lima, Peru; 5 Department of International Health, Bloomberg School of Public Health, John Hopkins University, Baltimore, MD USA; 6 Parasite Immunology Laboratory, Universidad Peruana Cayetano Heredia, Lima, Peru; 7 Department of Biomedical Sciences, Institute of Tropical Medicine, Antwerp, Belgium; 8 School of Public Health, Oregon Health and Science University-Portland State University, Portland, OR USA

**Keywords:** Ag-ELISA, Monoclonal antibodies, TsW8/TsW5, *Taenia solium*, *Taenia hydatigena*, Concordance, Cysticercosis, Cross-reaction

## Abstract

**Background:**

Antigen detection in *Taenia solium* cysticercosis confirms viable infection in the intermediate host (either pig or human). The reference B158/B60 monoclonal antibody (mAb)-based Ag-enzyme-linked immunosorbent assay (ELISA) has acceptable levels of sensitivity and specificity in human neurocysticercosis with multiple brain cysts, although its sensitivity is lower in cases with single brain cysts, whereas in porcine cysticercosis the assay specificity is affected by its frequent cross-reaction with *Taenia hydatigena*, another common cestode found in pigs. Our group has produced 21 anti-*T. solium* mAbs reacting against antigens of the whole cyst, vesicular fluid, and secretory/excretory products, identifying TsW8/TsW5 as the most promising pair of mAbs for an Ag-ELISA.

**Methods:**

We report the use of the TsW8/TsW5 Ag-ELISA to measure cysticercus antigen levels [expressed as optical density (OD) values] in two panels of sera collected from day 0 (baseline) to day 90 postinfection (PI) from pigs experimentally infected with *T. solium* (*n* = 26) and *T. hydatigena* (*n* = 12). At baseline and on days 28 and 90 PI, we used Bland–Altman (BA) analysis and Lin’s concordance correlation coefficients (CCC) to determine the concordance between the TsW8/TsW5 and the B158/B60 Ag-ELISA.

**Results:**

The TsW8/TsW5 Ag-ELISA was able to efficiently measure circulating antigen levels in *T. solium*-infected pigs, similar to that obtained with the B158/B60 Ag-ELISA. Almost all paired log-OD differences between assays were within the limits of agreement (LoA) in the BA analysis at baseline and on days 28 and 90 PI (92.3%, 100%, and 100%, respectively), and a high concordance of log-ODs between assays was also found (Lin’s CCC: 0.69, 0.92, and 0.96, respectively, all *P* < 0.001). In pigs infected with *T. hydatigena*, almost all paired log-OD differences were within the LoA in the BA analysis, whereas the concordance of log-ODs between assays was low at baseline (Lin’s CCC: 0.24) but increased on days 28 and 90 PI (Lins’ CCC: 0.88 and 0.98, *P* < 0.001).

**Conclusions/significance:**

The TsW8/TsW5 Ag-ELISA recognizes antigens in pigs with *T. solium* cysticercosis and is highly concordant with the B158/B60 Ag-ELISA. However, its diagnostic use is hampered by cross-reactions with *T. hydatigena*, as in other mAb-based Ag-ELISAs.

**Graphical Abstract:**

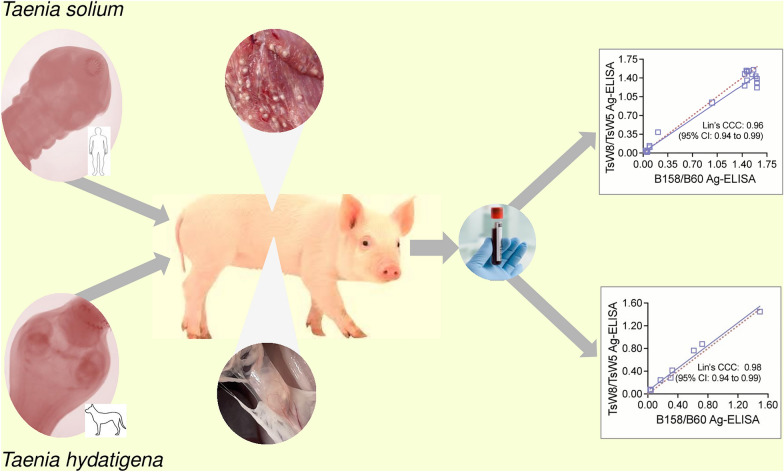

**Supplementary Information:**

The online version contains supplementary material available at 10.1186/s13071-024-06197-6.

## Background

*Taenia solium* is a zoonotic cestode in which humans are hosts of the adult stage (taeniasis) and pigs (as well as humans) are hosts of the intermediate stage (cysticercus) [1–3]. *Taenia solium* is endemic in many low- and middle-income countries (LMIC) that are characterized by poor sanitation and where pigs roam freely for food, increasing the risk of ingesting *T. solium* eggs and developing cysts in their musculature [4, 5]. Neurocysticercosis (NCC), the cyst infection of the central nervous system (CNS) is considered the main cause of human acquired epilepsy worldwide [6, 7], whereas porcine cysticercosis is an indicator of the level of environmental contamination with *T. solium*, and also affects the economy of farmers due to the confiscation of infected pork [1, 8]. On the other hand, *Taenia hydatigena* is a non-zoonotic cestode in which canids are definitive hosts of intestinal tapeworms and pigs serve as intermediate hosts of larval cysts that establish in the omentum and liver tissue and is often co-endemic with *T. solium* cysticercosis [9–12].

Antigen detection in *T. solium* cysticercosis is useful to confirm the presence of viable infection in the intermediate host (either pig or human) [13, 14]. In human NCC, antigen detection assays are preferably used to monitor the efficacy of antiparasitic treatment (measured in terms of reduction in circulating antigen levels) [13, 15], whereas in porcine cysticercosis antigen detection assays have been used in many research studies to estimate the burden of viable infection in porcine populations, and in control programs they would allow to rule out viable infection based in its high negative predictive value [16, 17].

The reference assay for antigen detection in cysticercosis is the capture antigen enzyme-linked immunosorbent assay (Ag-ELISA) using the set of monoclonal mAbs B158C1110 and B6084A4 (B158/B60) initially produced against *Taenia saginata* excretory/secretory antigens [18] and later adapted for human and porcine cysticercosis due to its genus-specific cross-reactivity with *T. solium* [17, 19, 20]. Currently, the B158/B60 Ag-ELISA is commercially available (apDIA®, Belgium) and is 99% specific and 94% sensitive in NCC cases with multiple cysts, according to the manufacturer, although its sensitivity is lower in cases with single cysts [13, 21]. In porcine cysticercosis, the specificity of the B158/B60 Ag-ELISA is very low (~ 50%) due to its frequent and intense cross-reaction with *T. hydatigena* as reported in previous studies [17, 20]. Another assay, the HP10 Ag-ELISA using the pairs of mAbs HP10/HP6 reacting against vesicular fluid antigens from *T. saginata* cysts, and also cross-reacting with homologous epitopes in *T. solium* cysts has been reported in the literature but only in research settings, with acceptable levels of sensitivity and specificity in human NCC (84% and 74% respectively) [15], although this assay also recognizes similar epitopes in other cestodes such as *T. hydatigena* [22].

Our group has previously produced a set of 21 anti-*T. solium* mAbs directed against cysticercal antigens from the whole cyst (TsW), vesicular fluid (TsV), and secretory/excretory (TsE) products [23]. Immunofluorescence and immunohistochemistry assays in histological sections from viable cysts showed reactivity of four mAbs to the vesicular space, nine mAbs reacted to the neck and cyst wall, one to the neck and vesicular space, and seven mAbs to the neck, cyst wall, and vesicular space [23]. Eight of these mAbs also detected parasite antigens in selected serum and urine samples from confirmed NCC cases [23]. The most promising pair of mAbs, TsW8 and TsW5, has been selected and adapted to a sandwich Ag-ELISA format using TsW8 as capture antibody and biotin-labeled TsW5 as detection antibody. To ascertain the potential of the TsW8/TsW5 Ag-ELISA as a novel diagnostic tool for antigen detection in cysticercosis, aspects such as reactivity against *T. solium* antigens, possible cross-reaction with *T. hydatigena* antigens, and its degree of concordance with the reference B158/B60 Ag-ELISA need to be addressed. Therefore, this study reports the concordance between the TsW8/TsW5 Ag-ELISA and the B158/B60 Ag-ELISA for measuring parasite-specific antigen levels in sera of pigs experimentally infected with *T. solium* and *T. hydatigena*.

## Methods

### Study design

This comparative study assessed the concordance between the TsW8/TsW5 Ag-ELISA and the reference B158/B60 Ag-ELISA for measuring cysticercal antigen levels in two panel of sera from pigs experimentally infected with *T. solium* and *T. hydatigena*, obtained from our bank of biological specimens.

### Samples

#### *Taenia solium*

Sera from pigs experimentally infected with *T. solium* were obtained from three previous studies using different infection schemes. The first study involved 18 pigs orally infected with a single gravid proglottid at 1, 3, and 5 months old (six pigs in each group) [24]. The second study involved four pigs orally infected with two gravid proglottids at 2 months old (data unpublished). We also included four pigs from a third study, in which 2-month-old pigs were infected with 10,000 activated oncospheres by intracarotid injection [25]. Tapeworms used in these experiments were obtained from patients treated with a single oral dose of 2 g of niclosamide [26]. Tapeworms were kept in transport medium with antibiotics (amphotericin B 0.25 µg/mL, streptomycin 100 µg/mL, and penicillin 10 IU/mL) and was adjusted to a volume of 10 mL of saline at 4 °C. *Taenia solium* was differentiated from *T. saginata* by morphological assessment of the scolex and gravid proglottids and confirmed by polymerase chain reaction (PCR) assay and partial cytochrome C oxidase subunit 1 (*COX1*) gene sequencing [27].

#### *Taenia hydatigena*

Sera were obtained from 12 two-month-old pigs experimentally infected with three different inocula of *T. hydatigena* eggs (5000, 10,000, and 20,000 eggs, respectively, four pigs each). Tapeworm specimens were obtained from adult dogs after arecoline purge (4 mg/kg) and collected in falcon tubes with transport medium at 4 °C. Differential diagnosis of *T. hydatigena* from other *Taenia* spp. in dogs was performed by morphometric assessment of rostellar hooks and molecular confirmation by PCR and partial *COX1* gene sequencing [27]. Eggs were obtained from gravid proglottids by gentle homogenization and assessed for viability by oncosphere activation and Trypan blue staining (a minimum viability percentage of 60% was optimal). We used immunofluorescence cavity slides for egg counting, and doses of 5000 eggs were prepared, resuspended in 180 µL of olive oil, and placed in gelatine capsules for immediate use of no longer than 10 min. Pigs were induced into anesthesia for experimental infection by injecting a mixture of ketamine (30 mg/kg) and xylazine (2 mg/kg) intramuscularly. An orogastric tube was immediately introduced through the pig’s mouth into the stomach, and capsules were pushed into the stomach using a syringe filled with saline solution (the number of capsules given per pig depended on the infection dose).

All pigs used in the experimental infections with *T. solium* and *T. hydatigena* belonged to commercial mixed-breed lines. Pigs were also kept in appropriate experimental conditions, housed in individual cages of 1 m × 1 m, with 12/12-h day/night cycles, with an average temperature of 21 °C, and received commercial feed and water ad libitum.

In all experiments, blood samples were taken from the cava vein of pigs at baseline (day 0), and on days 7, 14, 21, 28, 42, 56, 70, and 90 postinfection (PI). Blood samples were centrifuged, and 2 mL aliquots of serum were obtained and stored at –20 °C until processed.

### Assays

Sera were processed by the TsW8/TsW5 Ag-ELISA and the B158/B60 Ag-ELISA for the calculation of OD values (expressed in nanometers). Samples were analyzed in duplicate in each assay and the presence of discordant results between duplicates (e.g., OD readings with more than 15% of variation) were resolved by sample reprocessing. Laboratory technicians in charge of assays were masked to pig information (type of infection, postinfection time, and necropsy results). Plates used in each assay also included two well-defined control lines: positive (pool of sera from eight pigs with cysticercosis confirmed by necropsy exam), and negative (pool of sera from 8 pigs from commercial farms from a non-endemic *T. solium* area).

#### TsW8/TsW5 Ag-ELISA

We followed the procedures previously described for antigen detection in human NCC [23], but with some modifications for use on pig sera. Briefly, 96-well microtiter plates (MAXISORP flat bottom, Sigma-Aldrich®, St. Louis, MO, USA) were sensitized and incubated with purified TsW8 mAbs (2 µg/mL) diluted in carbonate-bicarbonate buffer for 1 h at 37 °C. Plates were washed once with PBS–Tween 0.05% in an automated microplate washer (ELX 50 TS, Biotek®, Vermont, USA). A blocking solution was added to each well and incubated for 30 min at 37 °C. The content was discarded and 100 µL/well of sera pretreated with 5% TCA and diluted at 1:6 was added, incubated another 30 min and then plates were washed six times. A total of 100 µL of biotinylated TsW5 mAbs (2 µg/mL, using the ABCAM-HRP Conjugation Kit – Lightning Link®) were added to each well. After 30 min of incubation, microtiter plates were washed once and streptavidin–horseradish peroxidase (HRP) (Jackson ImmunoResearch Laboratories, Ely, UK) diluted at 1:10,000 was added to each well and kept in incubation. Finally, 100 µL per well of O-phenylenediamine (OPD, Sigma-Aldrich®, St. Louis, MO, USA) diluted in citrate buffer was added as developer solution and stopped using H_2_SO_4_. ELISA plates were processed in a spectrophotometer at a wavelength of 490/650 nm to calculate the OD values (nm). We also divided the OD values of each sample with the OD from the negative controls to obtain antigen ratios (continuous variable). We arbitrarily considered a positive result for antigen detection if the antigen ratio was > 1.

#### B158/B60 Ag-ELISA

Sera were also processed by the B158/B60 Ag-ELISA for detection of cysticercal antigens according to the methodology previously described by Brandt and modified by van Kerckhoven [18, 19]. Briefly, 100 µL of B158C11A10 mAbs were added to each well of microtiter plates and kept in incubation for 30 min at 37 °C. Plates were washed with PBS–Tween 0.05% using an automated microplate washer, and then plates were blocked with 150 µL per well of a blocking solution (PBS–Tween 0.05% NBCS 1%). Subsequently, 100 µL of pretreated sera with TCA 5% diluted at 1:4 was added to each well of the microplates and kept in incubation for 15 min at 37 °C. After another washing step, 100 µL of biotinylated B60H8A4 mAbs were added and incubated for 15 min at 37 °C. An additional washing step was performed, and then 100 µL of streptavidin–HRP diluted at 1:10,000 was added and kept in incubation. A solution of OPD with citrate buffer and streptavidin peroxidase was added and the enzymatic reaction was stopped with H_2_SO_4_. OD values, antigen ratios, and positive results were also obtained as described above.

### Necropsy

Findings in pigs experimentally infected with *T. solium* at necropsy included the presence and number of viable and degenerated cysts by macroscopic evaluation in their carcasses (skeletal muscle, heart, tongue, and brain) [28, 29]. Findings in pigs experimentally infected with *T. hydatigena* at necropsy included the presence and number of viable cysts by macroscopic evaluation of the omentum, mesentery, and liver. Smaller caseous lesions suspected to be degenerated *T. hydatigena* cysts were confirmed by PCR assay and sequencing [27].

### Statistical analysis

Infection outcomes with *T. solium* and *T. hydatigena* in pigs were described using summary statistics [percentages for categorical variables; median and interquartile ranges (IQR) for numerical variables]. Ag-ELISA results (OD values, antigen ratios, and positive results) during the infection course with *T. solium* and *T. hydatigena* in pigs (from baseline to day 90 PI) were also summarized. For concordance analysis of assays, we used raw OD values since antigen ratios can be systematically different between assays due to intrinsic differences in OD values from negative controls used to calculate antigen ratios. OD values were transformed to natural logarithm scale to approximate these values to the normal distribution. Concordance analysis was performed separately on sera of from animals experimentally infected with *T. solium* and *T. hydatigena*, respectively. We used BA analysis to determine mean bias and LoA [mean bias ± 1.96 standard deviations (SD)] of paired log-OD differences between assays at baseline and on days 28 and 90 PI. We reported the percentage of paired log-OD differences between assays that were found within the LoA. Proportional bias in the BA analysis was assessed using a linear regression between paired log-OD differences and the average log-OD values between assays at each follow-up timepoint. Also, Lin’s concordance correlation coefficients (CCC) were used to calculate the level of concordance of paired log-OD values between assays using the same time points described above. Lin’s CCCs were calculated with their corresponding 95% confidence intervals. Finally, the correlation between parasite loads (number of viable cysts) in pigs at necropsy with the OD values measured with the TsW8TsW5 Ag-ELISA and the B158/B60 Ag-ELISA was analyzed using Spearman’s rank correlation coefficients. All the statistical analyses were carried out using the software GraphPad Prism version 9.5.1 (GraphPad software, LLC), considering a 5% significance level.

## Results

### Pigs experimentally infected with *T. solium*

From 26 pigs experimentally infected with *T. solium*, 17 (65.4%) had viable cysts at necropsy [median number of viable cysts: 257 (IQR: 198–556)]. Viable cysts were found in all pigs infected with either two gravid proglottids or 10,000 activated oncospheres, in 4/6 (66.7%) pigs that were infected with a single proglottid at 1 and 3 months old respectively, and only in one pig that was infected with a single gravid proglottid at 5 months old. Viable cyst burden was higher in pigs infected with a single proglottid at one month old (Table 1). On the other hand, degenerated cysts were found in 19/26 pigs [73.1%, median number of degenerated cysts: 151 (IQR: 36–831)], of which six pigs had only degenerated cysts (one and five pigs were infected with a single proglottid at 3 and 5 months old respectively). Pigs infected with a single proglottid at 5 months old had the highest median number of degenerated cysts [median: 714 (IQR: 582–1724)]. Three pigs did not have any type of cysts at necropsy (two pigs were infected with a single proglottid at 1 month old, and one pig was infected at 3 months old, Table 1).

**Table 1:** Necropsy findings in pigs experimentally infected with *T. solium* and *T. hydatigena*

Tapeworm used	Infection schemes	No. pigs	Viable cysts		Degenerated cysts	
			*n* (%)	Median (IQR)	%	Median (IQR)
*T. solium* (*n* = 26)	Single gravid proglottid by oral route at 1 month	6	4 (66.7)	2620 (1029–4753)	4 (66.7)	64 (20–141)
	Single gravid proglottid by oral route at 3 months	6	4 (66.7)	230 (198–394)	5 (83.3)	83 (25–139)
	Single gravid proglottid by oral route at 5 months	6	1 (16.7)	105 (-)	6 (100.0)	714 (582–1724)
	Two gravid proglottids by oral route at 2 months	4	4 (100.0)	457 (349–662)	4 (100.0)	269 (91–883)
	1000 activated oncospheres by intracarotid route at 2 months	4	4 (100.0)	217 (170–314)	0 (0.0)	ND
	TOTAL	26	17 (65.4)	257 (198–556)	19 (73.1)	151 (36–831)
*T. hydatigena* (*n* = 12*)	5000 eggs in gelatine capsules by oral route at 2 months	4	2 (50.0)	1 (−)	0 (0.0)	ND
	10,000 eggs in gelatine capsules by oral route 2 months	3	1 (33.3)	2 (−)	0 (0.0)	ND
	20,000 eggs in gelatine capsules by oral route at 2 months	4	1 (33.3)	1 (−)	0 (0.0)	ND
	TOTAL	11	4 (36.4)	1 (1–2)	0 (0.0)	ND

*IQR* interquartile range, *ND* not determined

^*^One pig infected with 10,000 *T. hydatigena* eggs died at day 28 postinfection

**Fig. 1:**
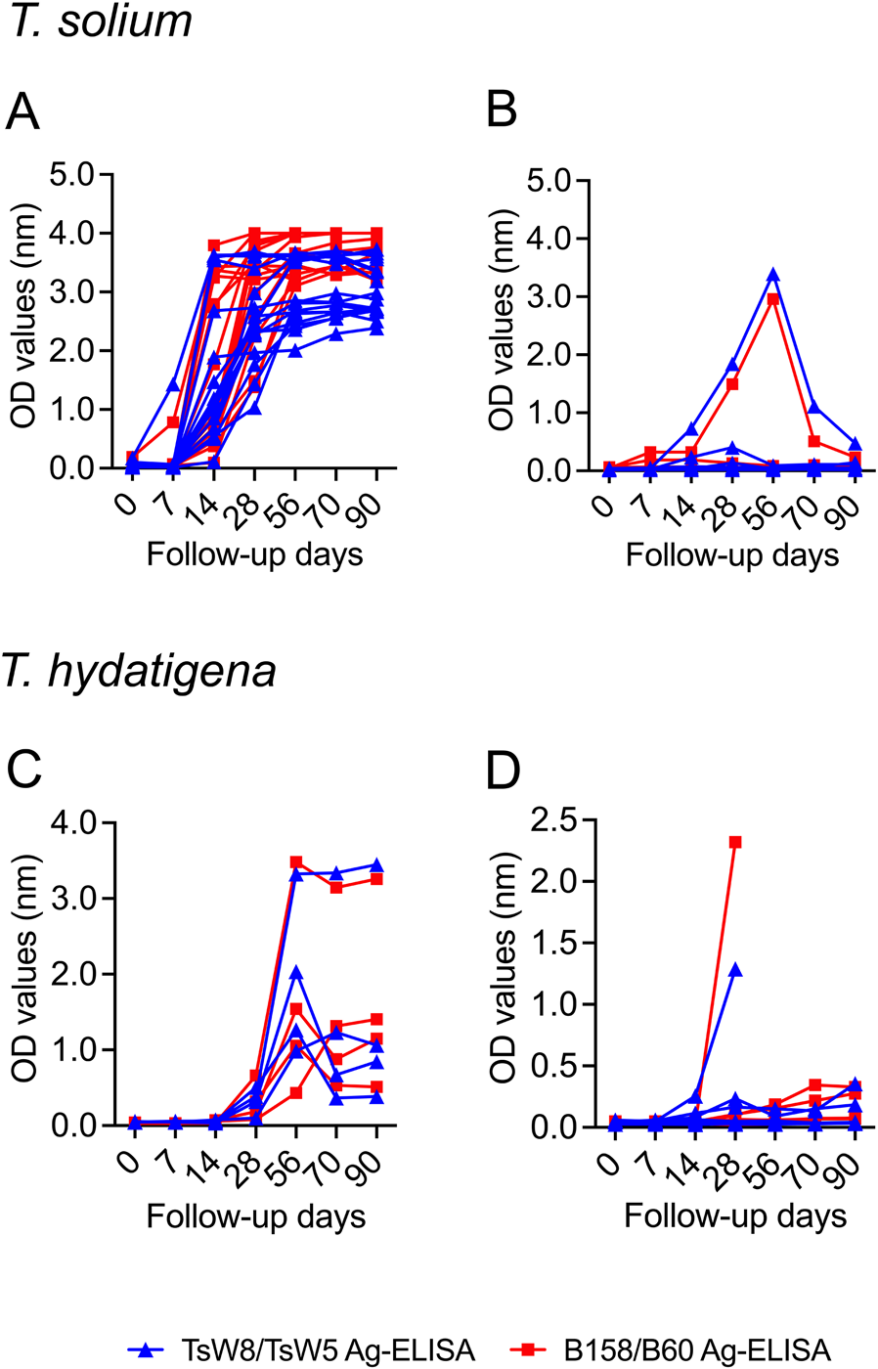
Antigen levels (OD values, in nanometers) measured with the TsW8/TsW5 Ag-ELISA (blue) and the B158/B60 Ag-ELISA (red) during the follow-up of pigs experimentally infected with *T. solium* (**A** pigs with viable cysts, *n* = 17; **B** pigs without viable cysts, *n* = 9) and *T. hydatigena* (**C** pigs with viable cysts, *n* = 4; **D** pigs without viable cysts, *n* = 8)

The TsW8/Ts5 Ag-ELISA was able to efficiently recognize circulating antigens in the pig sera during the course of infection with *T. solium*, observing a marked increase in OD values from baseline to day 28 PI in all pigs with viable cysts (*n* = 17) and reaching plateau levels until day 90 PI (all pigs were positive on TsW8/TsW5 Ag-ELISA with high ODs and antigen ratios, see Fig. 1A and Additional file 1: Table S1), with a similar performance to that obtained with the B158/B60 Ag-ELISA (Fig. 1A). In pigs without viable cysts (*n* = 9), the antigen levels (OD values and antigen ratios) measured with the TsW8/TsW5 Ag-ELISA were very low from baseline to day 14 PI in all pigs,and showed an increase at days 28 and 56 PI but subsequently decayed at days 70 and 90 PI in the subgroup of pigs with only degenerated cysts (*n* = 6), whereas in pigs without any type of cysts (*n* = 3) antigen levels were very low during all the follow-up (Fig. 1B). These profiles of antigen responses in pigs without viable cysts were similar for the B158/B60 Ag-ELISA (Fig. 1B, and Additional file 1: Table S1).

**Fig. 2:**
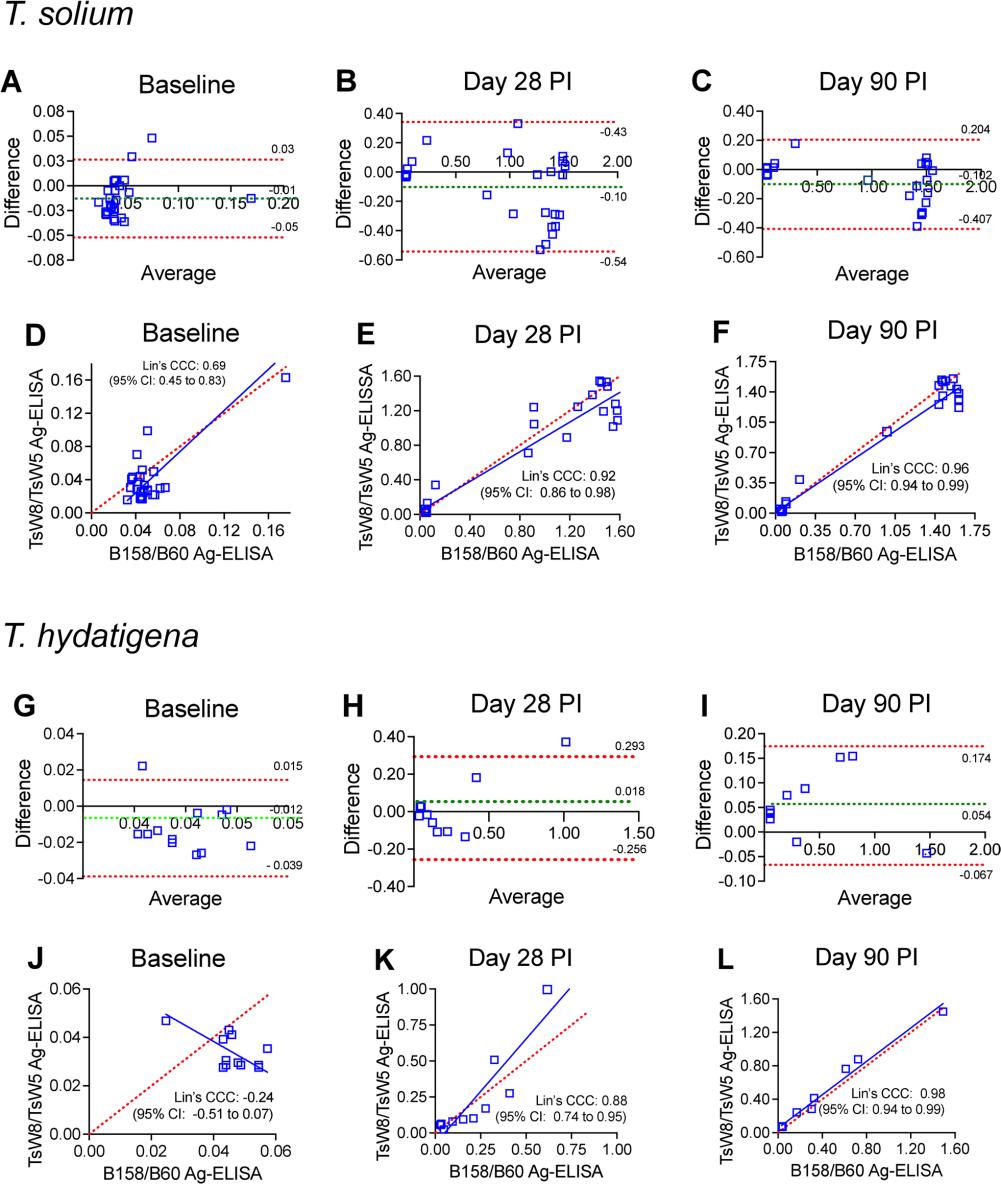
BA analysis and Lin’s CCC obtained from paired log-OD values between Ag-ELISAs in sera of pigs experimentally infected with *T. solium* at baseline, and at days 28 and 90 PI. The dotted red lines in the BA plots (**A**, **B**, **C**, **G**, **H**, **I**) show the LoA, and the dotted green lines show the mean bias. The solid blue lines in the scatter plots (**D**, **E**, **F**, **J**, **K**, **L**) represent the best-fit regression line between log-OD values of the TsW8/TsW5 Ag-ELISA and the B158/B60 Ag-ELISA, and the dotted red lines represents the lines of perfect concordance between assays

The results of the BA analysis between Ag-ELISAs are shown in Fig. 2. In general, all paired log-OD differences between assays were very close to the line of perfect agreement. At baseline, 92.3% (24/26) of the paired log-OD differences between assays were within the LoA (mean bias ± SD: −0.01 ± 0.02, Fig. 2A); two paired log-OD differences were outside the LoA and corresponded to two pigs with higher measurements with the TsW8/TsW5 Ag-ELISA versus the B158/B60 Ag-ELISA at baseline, whereas the paired log-OD difference on the right side in the BA plot corresponded to a pig with very high ODs measured with both Ag-ELISAs at baseline (Fig. 2A). The BA analysis at days 28 and 90 PI showed that 100% of the paired log-OD differences between assays were within the LoA (mean bias ± SD: 0.10 ± 0.23, and mean bias ± SD: −0.10 ± 0.16, respectively, Fig. 2B and Fig. 2C). The dispersion of log-OD differences visualized in the BA plots was proportional to the average log-OD values of both Ag-ELISAs, and proportional bias was not significant at baseline, but was statistically significant at days 28 and 90 PI (see Additional file 1: Table S2). Also, Lin’s CCC of paired log-ODs between Ag-ELISAs showed increased concordance during the follow-up of pigs, being only moderate at baseline (Lin’s CCC = 0.69 [95% CI: 0.45–0.83], *P* < 0.001, Fig. 2D), but higher at days 28 (Lin’s CCC = 0.92 [95% CI: 0.86–0.98], *P* < 0.001, Fig. 2E) and 90 PI (Lin’s CCC = 0.96 [95% CI: 0.94–0.99], *P* < 0.001, Fig. 2F).

We observed a higher correlation between viable cyst burden in pigs with OD values measured with the TsW8/TsW5 Ag-ELISA (Spearman’s *r* = 0.75, *P* < 0.001) compared with OD values measured with the B158/B60 Ag-ELISA (Spearman’s *r* = 0.68, *P* < 0.001).

### Pigs experimentally infected with *T. hydatigena*

From 12 pigs experimentally infected with *T. hydatigena* eggs, four (33.3%) had viable cysts at necropsy (two pigs were infected with 5000 eggs, and the other two pigs were infected with 10,000 and 20,000 eggs, respectively, Table 1). All cysts were found in the omentum, and in few numbers (only one or two cysts per pig). On the other hand, small, massive granulomas were found in the liver of a pig that died on day 28 PI. However, PCR did not confirm that these lesions were *T. hydatigena* cysts.

Antigen responses during the follow-up of pigs infected with *T. hydatigena* are shown in Fig. 1 and summarized in Additional file 1: Table S1. In the pigs with viable cysts (*n* = 4), antigen responses using either the TsW8/TsW5 Ag-ELISA or the B158/B60 ELISA were negative at baseline and at day 7 PI; two pigs were positive on B158/B60 Ag-ELISA at day 14 PI (although with low OD values and antigen ratios), whereas all pigs remained negative on TsW8/TsW5 Ag-ELISA. All pigs were positive using both Ag-ELISAs at day 28 PI and reached the highest OD values and antigen ratios at day 56 PI and onward (Fig. 1C). In the pigs without viable cysts (*n* = 7) antigen responses at baseline and at day 7 PI were negative using both Ag-ELISAs, whereas at day 14 PI three pigs were positive on B158/B60 Ag-ELISA (low OD values and antigen ratios) but no pigs were positive on TsW8/TsW5 Ag-ELISA. At day 28 PI and onward, only two pigs were positive in both Ag-ELISAs, with very low and similar OD values between assays (Fig. 1D).

The BA analysis showed that 92% (11/12) of the paired log-OD differences between Ag-ELISAs in pigs at baseline were within the LoA (mean bias ± SD: 0.01 ± 0.01, Fig. 2G). A similar percentage of agreement between assays in the BA analysis was found at day 28 PI (mean bias ± SD: 0.02 ± 0.14, Fig. 2H), whereas at day 90 PI all paired log-OD differences between assays were within the LoA (mean bias ± SD: 0.05 ± 0.06, Fig. 2I). Proportional bias in the BA analysis was only statistically significant at day 28 PI (see Additional file 1: Table S2). Finally, Lin’s CCC of paired log-ODs between Ag-ELISAs at baseline was not different from zero (Fig. 2J) but showed statistically higher concordance at day 28 (Lin’s CCC: 0.88 [95% CI: 0.74–0.95], *P* < 0.001, Fig. 2K) and at day 90 PI (Lin’s CCC: 0.98 [95% CI: 0.94–0.99], *P* < 0.001, and Fig. 2L).

## Discussion

Monitoring active infection in human NCC and porcine cysticercosis depends on the detection of circulating antigens in serum [13] by the current mAb-based Ag-ELISAs, which cross-react with *T. saginata* antigens. Our results showed that our TsW8/TsW5 Ag-ELISA recognizes antigenic epitopes in sera of pigs experimentally infected with *T. solium*, showing high levels of concordance (> 95%) with the reference B158/B60 Ag-ELISA, constituting the first report in demonstrated utility of this new assay for human and porcine cysticercosis, even though its use is hampered by the marked cross-reaction with *T. hydatigena* antigens as reported with other current mAb-based assays.

In general, antigen profiles during the follow-up of pigs experimentally infected with *T. solium* were very similar between assays. We observed high antigen levels in all pigs with viable cysticercosis at day 28 PI and onward, similar to previous studies reported in the literature [24, 30, 31]. Equally, the profile of antigen dynamic in pigs without cysticercosis infection or with only degenerated cysts also showed a similar trend between assays.

Although a lower concordance level between Ag-ELISAs was observed at baseline, this increased over time in pigs infected with *T. solium*. A possible explanation for this could be the intrinsic differences from each assay since almost all infected pigs were antigen negative at baseline, and the pig that presented high antigen levels at the baseline, in both assays, may suggest a preexisting infection with either *T. solium* or *T. hydatigena* detected with both assays.

Concordance in OD readings between assays was higher at days 28 PI and 90 PI (Lin’s CCC > 0.90). Similar levels of concordance between Ag-ELISAs have been reported in a recent cross-sectional study using sera from patients with parenchymal and subarachnoid NCC [32], which corroborates the potential of our TsW8/TsW5 Ag-ELISA for antigen detection in cysticercosis. Proportional bias was observed in BA analysis during the follow-up of pigs with a positive trend in OD differences with higher average measurement of OD values. This phenomenon can indicate differences in the avidity of mAbs between assays when the amount of antigen detection is high. While mAbs B158/B60 recognize excretory/secretory products (~67 kDa) released from cysts [18, 33], our TsW8/TsW5 mAbs recognize surface structure components of the neck and cyst wall and seems to have higher sensitivity at initial days of infection or when cysts are degenerated. Since we selected a panel of sera from *T. solium*-experimentally infected pigs with high cyst burden, we cannot determine whether the correlation between Ag-ELISAs in porcine cysticercosis is maintained in samples from pigs with moderate or low cyst burden.

We also observed cross-reactivity of our TsW8/TsW5 Ag-ELISA with *T. hydatigena* cyst antigens, with apparently similar levels to that observed for the B158/B60 Ag-ELISA [16, 17, 20]. Although the previous study of Paredes et al. [23] did not report that the mAbs TsW8/TsW5 cross-react with antigens from the vesicular fluid of *T. hydatigena* cysts [23], this does not necessarily exclude the possibility of reaction against other antigenic components of the parasite as demonstrated in our study. However, cross-reaction with *T. hydatigena* is not a limitation for its potential use as a diagnostic tool for antigen detection in human NCC [32–34], given that this parasite only infects humans very exceptionally. On the other hand, the *T. hydatigena* cross-reaction of the TsW8/TsW5 Ag-ELISA in experimentally infected pigs limits its potential use to estimate *T. solium* infection in porcine populations in areas co-endemic with *T. hydatigena* [16]. Further studies need to be conducted to properly evaluate the assay specificity, especially with other common cestode infections in humans such as cystic echinococcosis, and with other tapeworm infections of pigs.

This study has some drawbacks. First, the relatively small sample size in both experiments (especially for the group of pigs experimentally infected with *T. hydatigena*) may affect the statistical power of our results. Second, the distribution of pigs experimentally infected with *T. solium* included pigs without viable infection and pigs with severe viable infection, so we were unable to assess the performance of the TsW8/TsW5 Ag-ELISA in the presence of pigs with low parasite loads. Third, positive/negative results using the TsW8/TsW5 Ag-ELISA were arbitrarily determined if sample OD readings were at least higher than OD readings of negative controls (antigen ratio > 1). However, future studies are required to determine the optimal cutoff point of the antigen ratio (e.g., using ROC analysis) to classify positive and negative results in naturally infected pigs for assessing test specificity and sensitivity. Finally, the antigen levels in some serum samples could have been affected (reduced) due to their longevity, although this would have affected both assays.

## Conclusions

Our findings showed a similar performance between our TsW8/TsW5 Ag-ELISA and the B158/B60 Ag-ELISA for assessing the antigen profiles in pigs during experimental infection with *T. solium*, being a promising tool for the follow-up of antigen dynamics in human NCC. Nevertheless, the use of our TsW8/TsW5 Ag-ELISA for antigen detection in porcine cysticercosis is hampered by its cross-reaction with *T. hydatigena* antigens, similar to that reported with previous mAb-based assays [11, 20, 22]. Further studies are required to properly assess the sensitivity and specificity of the TsW8/TsW5 Ag-ELISA in human NCC and porcine cysticercosis, and to evaluate other adaptations of the TsW8/TsW5 mAbs such as low-cost techniques for diagnostic use in low-resource settings.

### Supplementary Information

**Additional file 1: Table S1.** Summary statistics of Ag-ELISA results during the follow-up of pigs experimentally infected with *T. solium* and *T. hydatigena*. **Table S2.** Linear regression coefficients to assess proportional bias in the Bland–Altman analysis of paired log-ODs between the TsW8/TsW5 Ag-ELISA and the B158/B60 Ag-ELISA in sera of pigs experimentally infected with *T. solium* and *T. hydatigena*.

## Data Availability

Data supporting the conclusions of this study are included as associated file. Raw data are also available from the corresponding author under reasonable request.

## Abbreviations

NCC: Neurocysticercosis
Ag-ELISA: Antigen detection enzyme-linked immunosorbert assay
mAb: monoclonal-based antibody
BA: Bland-Altman
LoA: Limits of agreement
CCC: Concordance Correlation Coefficient
OD: Optical density
