# Supplementary material for: Concordance between two monoclonal antibody-based antigen detection enzyme-linked immunosorbent assays for measuring cysticercal antigen levels in sera from pigs experimentally infected with Taenia solium and Taenia hydatigena
Source: Parasit Vectors. 2024 Apr 2;17:172. doi: 10.1186/s13071-024-06197-6 (PMC10988810; doi:10.1186/s13071-024-06197-6)
Supplement: Supplementary file 1 — Additional file 1: Table S1. Summary statistics of Ag-ELISA results during the follow-up of pigs experimentally infected with T. solium and T. hydatigena. Table S2. Linear regression coefficients to assess proportional bias in the Bland–Altman analysis of paired log-ODs between the TsW8/TsW5 Ag-ELISA and the B158/B60 Ag-ELISA in sera of pigs experimentally infected with T. solium and T. hydatigena. [file 13071_2024_6197_MOESM1_ESM.docx]

**Additional file 1: Table S1**: Summary statistics of Ag-ELISA results during the follow-up of pigs experimentally infected with *T. solium* and *T. hydatigena*.

| Tapeworm used | Infection status | Follow-up days | TsW8/TsW5 Ag-ELISA | | | B158/B60 Ag-ELISA | | |  |
| --- | --- | --- | --- | --- | --- | --- | --- | --- | --- |
|  |  |  | OD values (mean ± SD) | Antigen ratios (mean ± SD) | Positive  (n, %) | OD values  (mean ± SD) | Antigen ratios (mean ± SD) | Positive  (n, %) |  |
|  |  |  |  |  |  |  |  |  |  |
|  |  |  |  |  |  |  |  |  |  |
| *T. solium* | Pigs with viable cysticercosis (*n* = 17) | 0 | 0.0 ± 0.0 | 0.9 ± 0.8 | 4 (23.5) | 0.1 ± 0.0 | 0.9 ± 0.7 | 1 (5.6) |  |
|  |  | 7 | 0.1 ± 0.3 | 2.2 ± 6.7 | 3 (17.7) | 0.1 ± 0.2 | 1.5 ± 3.3 | 1 (5.6) |  |
|  |  | 14 | 1.7 ± 1.3 | 30.1 ± 24.5 | 17 (100.0) | 1.7 ± 1.4 | 27.9 ± 22.7 | 17 (100.0) |  |
|  |  | 28 | 2.5 ± 0.8 | 47.2 ± 16.9 | 17 (100.0) | 3.2 ± 0.8 | 51.0 ± 11.9 | 17 (100.0) |  |
|  |  | 56 | 3.0 ± 0.6 | 59.0 ± 25.0 | 17 (100.0) | 3.7 ± 0.3 | 58.8 ± 7.4 | 17 (100.0) |  |
|  |  | 70 | 3.1 ± 0.5 | 60.2 ± 25.1 | 17 (100.0) | 3.7 ± 0.3 | 59.6 ± 7.6 | 17 (100.0) |  |
|  |  | 90 | 3.0 ± 0.4 | 59.1 ± 23.3 | 17 (100.0) | 3.7 ± 0.3 | 59.6 ± 7.6 | 17 (100.0) |  |
|  | Pigs without viable cysticercosis (*n* = 9) | 0 | 0.0 ± 0.0 | 0.7 ± 0.3 | 1 (11.1) | 0.0 ± 0.0 | 0.8 ± 0.1 | 0 (0.0) |  |
|  |  | 7 | 0.0 ± 0.0 | 0.6 ± 0.3 | 1 (11.1) | 0.0 ± 0.0 | 0.8 ± 0.1 | 0 (0.0) |  |
|  |  | 14 | 0.1 ± 0.2 | 3.1 ± 5.1 | 5 (55.6) | 0.1 ± 0.1 | 1.8 ± 2.0 | 4 (44.4) |  |
|  |  | 28 | 0.3 ± 0.6 | 6.1 ± 13.2 | 6 (66.7) | 0.2 ± 0.5 | 4.4 ± 10.3 | 3 (33.3) |  |
|  |  | 56 | 0.4 ± 1.1 | 9.2 ± 24.8 | 5 (55.6) | 0.4 ± 1.0 | 7.8 ± 20.7 | 4 (44.4) |  |
|  |  | 70 | 0.2 ± 04 | 3.7 ± 7.9 | 6 (66.7) | 0.1 ± 0.2 | 2.0 ± 3.3 | 4 (44.4) |  |
|  |  | 90 | 0.1 ± 0.1 | 2.2 ± 3.3 | 5 (55.6) | 0.1 ± 0.1 | 1.5 ± 1.4 | 4 (44.4) |  |
| *T. hydatigena* | Pigs with viable cysticercosis (*n* = 4) | 0 | 0.0 ± 0.0 | 0.4 ± 0.1 | 0 (0.0) | 0.1 ± 0.0 | 0.8 ± 0.1 | 0 (0.0) |  |
|  |  | 7 | 0.0 ± 0.0 | 0.4 ± 0.0 | 0 (0.0) | 0.1 ± 0.0 | 0.9 ± 0.1 | 0 (0.0) |  |
|  |  | 14 | 0.0 ± 0.0 | 0.6 ± 0.2 | 0 (0.0) | 0.1 ± 0.0 | 0.9 ± 0.3 | 2 (50.0) |  |
|  |  | 28 | 0.3 ± 0.2 | 3.8 ± 3.1 | 4 (100.0) | 0.3 ± 0.2 | 5.5 ± 2.8 | 4 (100.0) |  |
|  |  | 56 | 1.6 ± 1.3 | 19.9 ± 16.1 | 4 (100.0) | 1.9 ± 1.0 | 31.7 ± 17.4 | 4 (100.0) |  |
|  |  | 70 | 1.5 ± 1.2 | 17.9 ± 14.2 | 4 (100.0) | 1.4 ± 1.3 | 23.4 ± 22.3 | 4 (100.0) |  |
|  |  | 90 | 1.6 ± 1.2 | 19.3 ± 14.4 | 4 (100.0) | 1.4 ± 1.4 | 23.9 ± 22.9 | 4 (100.0) |  |
|  | Pigs without viable cysticercosis (*n* = 8) | 0 | 0.0 ± 0.0 | 0.4 ± 0.1 | 0 (0.0) | 0.0 ± 0.0 | 0.7 ± 0.2 | 0 (0.0) |  |
|  |  | 7 | 0.0 ± 0.0 | 0.4 ± 0.1 | 0 (0.0) | 0.0 ± 0.0 | 0.7 ± 0.2 | 0 (0.0) |  |
|  |  | 14 | 0.0 ± 0.0 | 0.5 ± 0.1 | 0 (0.0) | 0.1 ± 0.0 | 1.0 ± 0.5 | 3 (42.9) |  |
|  |  | 28* | 0.1 ± 0.0 | 0.8 ± 0.3 | 2 (28.6) | 0.1 ± 0.1 | 1.4 ± 1.4 | 2 (28.6) |  |
|  |  | 56 | 0.1 ± 0.1 | 1.0 ± 0.7 | 2 (28.6) | 0.1 ± 0.1 | 1.0 ± 0.8 | 2 (28.6) |  |
|  |  | 70 | 0.1 ± 0.1 | 1.6 ± 0.3 | 2 (28.6) | 0.1 ± 0.1 | 1.1 ± 0.9 | 2 (28.6) |  |
|  |  | 90 | 0.1 ± 0.1 | 1.7 ± 1.4 | 2 (28.6) | 0.1 ± 0.1 | 1.7 ± 2.1 | 2 (28.6) |  |

Abbreviations: OD (optical density); SD (standard deviation)

*One pig died at day 28 PI

**Additional file 2: Table S2**. Linear regression coefficients to assess proportional bias in the Bland-Altman analysis of paired log-ODs between the TsW8/TsW5 Ag-ELISA and the B158/B60 Ag-ELISA in sera of pigs experimentally infected with *T. solium and T. hydatigena*.

| Tapeworm used for experimental infection | Follow-up day | Intercept (95% CI) | Slope (95% CI) |
| --- | --- | --- | --- |
| *Taenia solium* | Baseline | -0.02 (-0.04 to -0.01) | 0.19 (-0.11 to 0.49) |
|  | Day 28 PI | 0.04 (-0.12 to 0.19) | -0.15 (-0.29 to -0.01) |
|  | Day 90 PI | 0.01 (-0.09 to 0.11) | -0.12 (-0.20 to -0.03) |
| *Taenia hydatigena* | Baseline | 0.02 (-0.08 to 0.13) | -0.85 (-3.49 to 1.79) |
|  | Day 28 PI | -0.05 (-0.14 to 0.02) | 0.36 (0.12 to 0.60) |
|  | Day 90 PI | 0.05 (-0.01 to 0.13) | -0.01 (-0.10 to 0.10) |

Abbreviations: OD (optical density), CI (confidence interval), PI (post-infection)
